# Evaluating feature extraction reproducibility across image biomarker standardization initiative‐compliant radiomics platforms using a digital phantom

**DOI:** 10.1002/acm2.70110

**Published:** 2025-05-12

**Authors:** Han‐Back Shin, Heesoon Sheen, Jang‐Hoon Oh, Young Eun Choi, Kihoon Sung, Hyun Ju Kim

**Affiliations:** ^1^ Department of Radiation Oncology Gachon University Gil Medical Center Incheon Republic of Korea; ^2^ Department of Health Sciences and Technology, Samsung Advanced Institute for Health Sciences & Technology Sungkyunkwan University Seoul Republic of Korea; ^3^ High‐Energy Physics Center Chung‐Ang University Seoul Republic of Korea; ^4^ Department of Radiology Kyung Hee University Hospital, Kyung Hee University College of Medicine Seoul Republic of Korea; ^5^ Department of Radiation Oncology, Gil Medical Center Gachon University College of Medicine Incheon Republic of Korea

**Keywords:** benchmarking procedures, IBSI‐compliant platforms, radiomics, reproducibility, standardization

## Abstract

**Background:**

The aim of this study was to thoroughly analyze the reproducibility of radiomics feature extraction across three Image Biomarker Standardization Initiative (IBSI)‐compliant platforms using a digital phantom for benchmarking. It uncovers high consistency among common features while also pointing out the necessity for standardization in computational algorithms and mathematical definitions due to unique platform‐specific features.

**Methods:**

We selected three widely used radiomics platforms: LIFEx, Computational Environment for Radiological Research (CERR), and PyRadiomics. Using the IBSI digital phantom, we performed a comparative analysis to extract and benchmark radiomics features. The study design included testing each platform's ability to consistently reproduce radiomics features, with statistical analyses to assess the variability and agreement among the platforms.

**Results:**

The results indicated varying levels of feature reproducibility across the platforms. Although some features showed high consistency, others varied significantly, highlighting the need for standardized computational algorithms. Specifically, LIFEx and PyRadiomics performed consistently well across many features, whereas CERR showed greater variability in certain feature categories than LIFEx and PyRadiomics.

**Conclusion:**

The study findings highlight the need for harmonized feature calculation methods to enhance the reliability and clinical usefulness of radiomics. Additionally, this study recommends incorporating clinical data and establishing benchmarking procedures in future studies to enhance the role of radiomics in personalized medicine.

## 1 INTRODUCTION

Radiomics is an innovative and rapidly evolving field in radiation oncology that utilizes advanced image analysis techniques to extract a comprehensive array of quantitative features from medical images.^1^, ^2^, ^3^, ^4^, ^5^ By revealing patterns and characteristics that are not easily discernible through conventional imaging assessment,^4^, ^6^ radiomics offers deeper insights into tumor heterogeneity, potentially enabling more precise patient stratification and prediction of treatment responses.^1^, ^7^ These insights are primarily achieved by integrating machine learning and advanced statistical methods capable of identifying subtle, high‐dimensional relationships in imaging data.^8^, ^9^, ^10^, ^11^, ^12^

Despite its significant potential for personalized cancer care, the clinical integration of radiomics is hampered by several challenges related to standardization and reproducibility.^6^, ^13^, ^14^ Variations in image acquisition protocols, preprocessing steps, and feature extraction methods frequently lead to inconsistencies, thereby limiting comparability and generalizability across different studies.^15^, ^16^, ^17^ In response to these issues, the Image Biomarker Standardization Initiative (IBSI) was established in 2015 to develop standardized protocols for radiomics analyses, with a particular emphasis on feature extraction procedures.^6^, ^16^

Although IBSI‐compliant methodologies have addressed some challenges, inter‐platform variability remains a significant issue.^18^, ^19^, ^20^ Differences in mathematical definitions, computational algorithms, and software implementations still result in disparate feature values, even among IBSI‐compliant platforms.^19^, ^21^ Paquier et al.^19^ demonstrated that IBSI adherence does not ensure concordance in feature values, comparing multiple radiomics software tools using digital phantoms and patient data. Similarly, Fornacon‐Wood et al.^22^ reported that the reliability and prognostic impact of radiomics features heavily depend on the feature extraction platform used.

To investigate this issue further, our study evaluates the reproducibility of radiomics feature extraction across three IBSI‐compliant software platforms: LIFEx, PyRadiomics, and CERR. Using a digital phantom for benchmarking, we systematically assess feature value consistency and identify potential sources of variability. This study extends previous research, emphasizing the need for standardized computational algorithms and mathematical definitions to support the reliable clinical utility of radiomics.

## 2 MATERIALS AND METHODS

### 2.1 Study design

To evaluate each software's ability to accurately and consistently extract radiomics features according to IBSI standards, we designed a comparison study using a digital phantom as a benchmarking tool.^6^, ^16^ Radiomics features were categorized into three groups, and statistical analyses were performed on features commonly extracted across all platforms. This approach provided insights into the reproducibility and consistency of feature extraction, thereby informing broader standardization efforts in radiomics.

**FIGURE 1:**
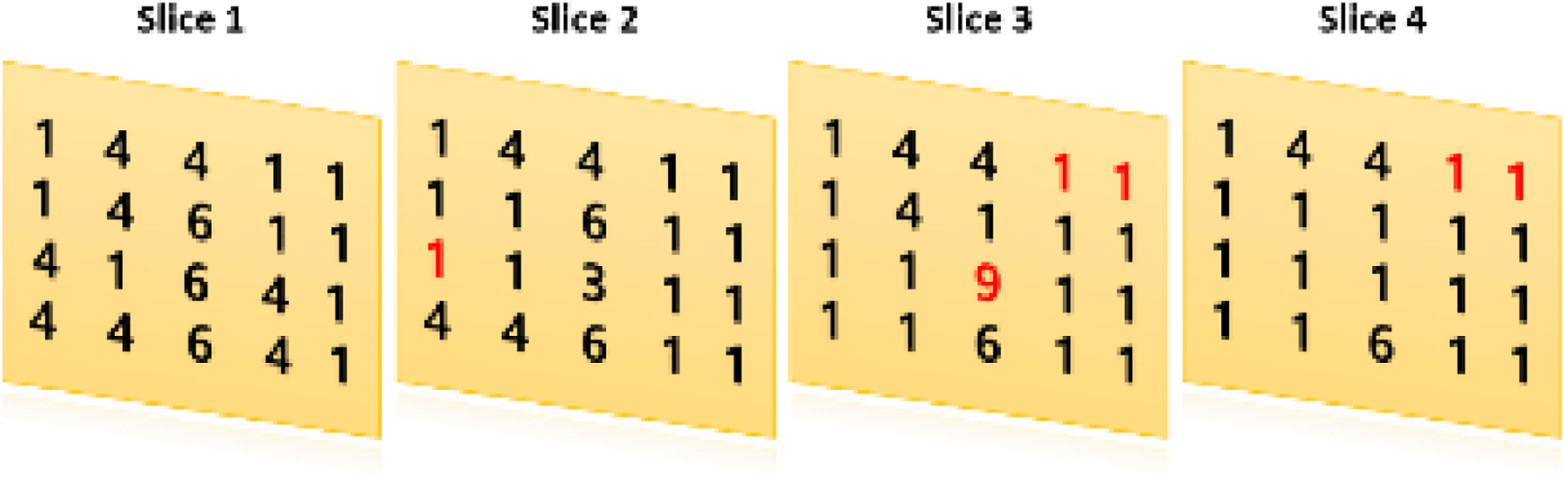
Axial slice of an IBSI digital phantom (5 × 4 × 4). The red color voxels are excluded from the mask to ensure feature calculations focus exclusively on analyzable areas. IBSI, Image Biomarker Standardization Initiative.

For feature evaluation, we used the IBSI digital phantom, recommended for radiomics quality assurance. The phantom is structured as a 5 × 4 × 4 voxel grid (each voxel measuring 2 × 2 × 2 mm^3^) with integer gray levels from 1 to 9.^6^, ^18^ As shown in Figure 1, the associated structure file highlights certain voxels in red, which are excluded from the region of interest (ROI) to ensure that feature calculations focus exclusively on analyzable areas, following IBSI guidelines.^6^

**FIGURE 2:**
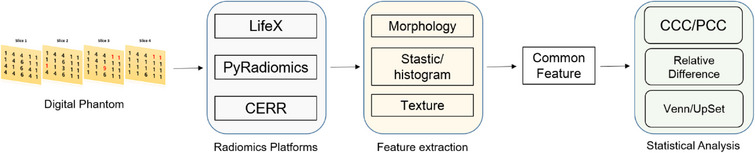
Overall workflow of inter‐radiomics platform evaluation. This figure illustrates the step‐by‐step process used to assess the reproducibility of radiomics feature extraction across the three platforms.

The IBSI digital phantom offers a standardized benchmarking tool that enables consistent validation of radiomics features across different imaging modalities, software platforms, and computational algorithms. This standardization is critical for ensuring the reproducibility of radiomics studies and the robustness of findings across various technical environments. Therefore, our study used a digital phantom along with the radiomics feature names and reference values provided by the IBSI to compare the extracted values. Figure 2 illustrates the workflow used to evaluate the accuracy and reproducibility of the assessments using a single IBSI‐introduced digital phantom and three radiomics software platforms.

### 2.2 Radiomics platforms

In this study, three widely used radiomics platforms (LIFEx, CERR, and PyRadiomics) were selected to extract radiomics features for comparative analysis (Table 1).^15^, ^21^, ^23^ Notable for its JAVA‐based development, LIFEx does not support model building, but offers IBSI compliance, built‐in segmentation, and a non‐open‐source policy.^21^ Despite the absence of radiomics maps, it features integrated visualization, making it user‐friendly. Its significant advantage lies in its accessibility to medical imaging professionals without requiring programming skills. LIFEx offers interactive index calculations that enhance reproducibility across centers. Its user‐friendly interface ensures consistent workflows by minimizing operator‐induced variability. Real‐time feedback mechanisms enable users to refine segmentations and parameter selections according to standardized criteria, thereby improving the consistency of extracted features. This interactivity also supports cross‐institutional benchmarking by aligning computational processes with IBSI‐compliant protocols. Consequently, discrepancies in radiomics data interpretation are minimized, facilitating reliable replication of results across both research and clinical settings. Recognized for its flexibility and Python‐based architecture, PyRadiomics excels in model building and IBSI compliance.^23^ Although it lacks built‐in segmentation and radiomics maps, its open‐source nature and standalone functionality, coupled with integration capabilities with 3D Slicer underscore its utility in advanced radiomics research. As an open‐source MATLAB‐based platform, CERR stands out for its model‐building capabilities, IBSI compliance, inclusion of radiomics maps, visualization, and built‐in segmentation.^15^ Its strengths lie in its comprehensive data management and visualization features, although programming knowledge is required for optimal use.

**TABLE 1:** Summary of key features of radiomics platforms.

	LIFEx	Pyradiomics	CERR
Programming language	JAVA	Python	MATLAB
Model building	No	No	Yes
IBSI‐compliant	Yes	Yes	Yes
Radiomics maps	No	Yes	Yes
Built‐in segmentation	Yes	No	Yes
Open‐source	No	Yes	Yes
Integrated visualization	Yes	No	Yes

Abbreviations: CERR, computational environment for radiological research; IBSI, Image Biomarker Standardization Initiative.

### 2.3 Three categories of IBSI‐standardized features

This study analyzed 215 features standardized by the IBSI across three radiomics platforms to evaluate the reproducibility of inter‐platform tests. The 215 radiomics features were divided into three categories, with each category's specific features and numbers detailed as follows.^18^

Of the 215 IBSI‐standardized features analyzed, the number of extracted features varied among platforms: 108 for LIFEx, 172 for CERR, and 120 for PyRadiomics. These variations reflect platform‐specific implementations, including unique classification methods, gray‐level binning strategies, and calculation techniques. For example, gray‐level size zone matrix (GLSZM) features were extracted exclusively by PyRadiomics, whereas neighboring gray‐level dependence matrix (NGLDM) features were computed only by CERR. As detailed in Tables S1–S3, the lists of all extracted features, their corresponding IBSI reference values, and platform‐specific results highlight these discrepancies, providing transparency and facilitating reproducibility. These differences emphasize the critical need for continued standardization efforts in radiomics. In the morphology category, which comprises 29 features, emphasis is placed on the shape characteristics of the ROI.^6^ These features are crucial for understanding the geometric aspects of a target area and offer valuable insights into its structural composition and boundaries. The Statistics/Histogram category consists of 50 features focused on analyzing variations in local intensity and distribution patterns observed in the intensity histograms. This section quantitatively assesses the variations in intensity throughout the imaging data, highlighting the texture‐related characteristics of the tissue. Finally, the texture category, which was the most comprehensive of the three, included 136 features. To ensure consistent feature extraction across all platforms, we selected a bin size of 1 to preserve the original image intensities, utilized B‐spline interpolation to minimize sampling artifacts, and set a gray‐level co‐occurrence matrix (GLCM) distance of 1 to effectively capture local texture information. Additionally, no resampled pixel spacing was applied, preserving the native image resolution. These parameters align with IBSI guidelines, thereby ensuring reproducibility and comparability of radiomics features across various software implementations.^6^, ^17^ It employs sophisticated texture analysis techniques, such as the GLCM, gray‐level run length matrix (GLRLM), GLSZM, neighboring gray‐tone difference matrix (NGTDM), and NGLDM, for in‐depth analysis of texture patterns present in the imaging data.

### 2.4 Statistical analyses

To methodically evaluate the reproducibility of radiomics features extracted across different software tools, we used a detailed analytical framework using RStudio version 2023.03.0. Venn diagrams were created to illustrate both shared and unique features extracted by the three software packages, offering a straightforward method for comparing their feature extraction capabilities. The UpSet plot technique was used to manage complex intersections and provide a quantitative overview of feature overlaps.^18^ Concordance correlation coefficients (CCCs) were used to determine the consistency between the features extracted by each software and the benchmark values provided by the IBSI. Pearson correlation coefficient (PCC) was used to measure the linear correlation between the features extracted using the software and IBSI standards.^17^ Spearman's rank correlation coefficient was used to assess the monotonic relationships among ranked feature values across different software programs relative to the IBSI references. We calculated the absolute relative differences (RDs)^6^ between the feature values extracted by each software and the IBSI reference values to gauge the extent of discrepancies. The RD values were then categorized into four levels of accuracy: excellent (RD ≤ 1%), good (1% < RD ≤ 5%), moderate (5% < RD ≤ 10%), and poor (RD > 10%), providing a structured assessment of agreement quality.^18^

## 3 RESULTS

This study comprehensively analyzed the reproducibility of radiomics feature extraction across three widely used IBSI‐compliant platforms (LIFEx, CERR, and PyRadiomics) for benchmarking using a single digital phantom.^19^, ^22^ Table 2 displays the counts of IBSI‐standardized features, along with the number of features extracted for each of the three platforms. In this study, 215 IBSI‐standardized features were analyzed, with 108 features extracted for LIFEx, 120 for PyRadiomics, and 172 for CERR. In the morphology category (29 features), LIFEx, PyRadiomics, and CERR extracted 12, 11, and 13 features, respectively. In the statistics and histogram categories (50 features), the extracted features were 41 for LIFEx, 39 for CERR, and 28 for PyRadiomics. Among the textural features (136 in total), the number of features were 55 for LIFEx, 120 for CERR, and 81 for PyRadiomics. Of the 215 features, 64 were extracted consistently and commonly across all platforms. As shown in Tables S1–S3, the lists of common features across platforms emphasize the consistency achieved under IBSI‐compliant protocols. Notably, in the Statistics/Histogram category, 27 common features demonstrated excellent reproducibility, as evidenced by their close alignment with IBSI reference values.

**TABLE 2:** Extracted feature numbers of three radiomics platforms.

Category	IBS‐standardized features	Common features	LIFEx	PyRadiomics	CERR
Morphology	29	5	12	11	13
Statistics/histogram	50	27	41	28	39
Texture	136	32	55	81	120
Sum	215	64	108	120	172

Abbreviations: CERR, computational environment for radiological research.

**FIGURE 3:**
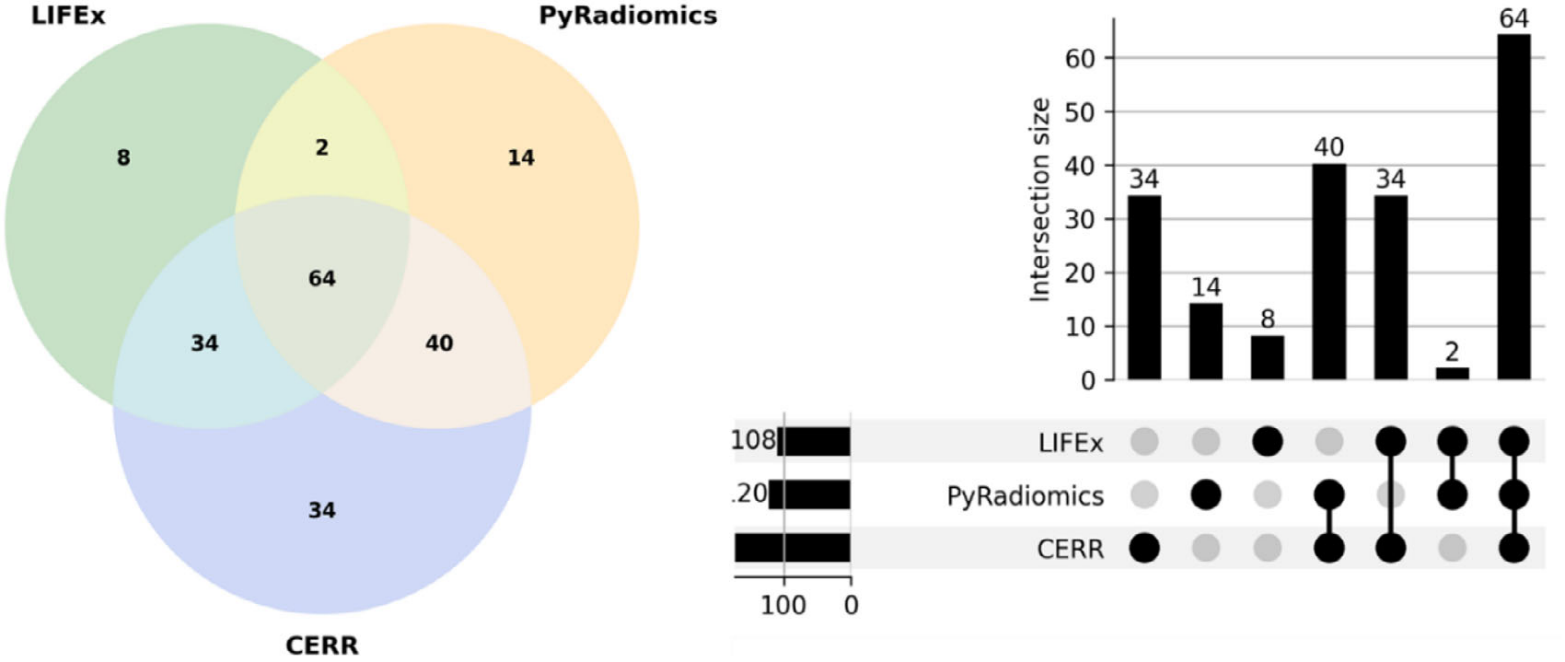
The Venn and UpSet diagram across radiomics platforms. This figure highlights the overlap and unique features extracted by the three IBSI‐compliant radiomics platforms, providing a visual comparison of feature reproducibility.

Figure 3 depicts a Venn and UpSet diagram illustrating shared and unique features across different platforms. The diagram shows 34 common features between LIFEx and CERR, 40 between PyRadiomics and CERR, and only 2 between LIFEx and PyRadiomics. Additionally, the analysis revealed platform‐specific features with no overlap with the others: 8 features unique to LIFEx, 14 unique to PyRadiomics, and 34 unique to CERR.

**FIGURE 4:**
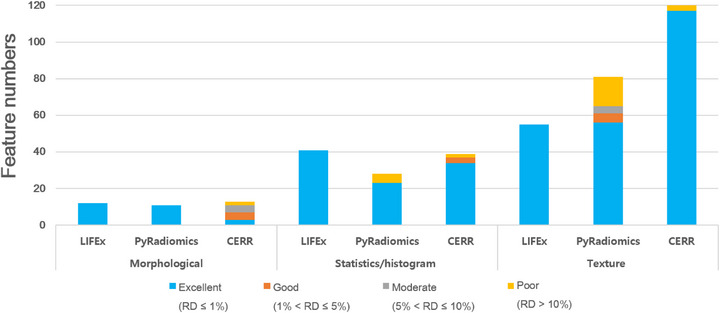
Degree of RD^6^ in categories across radiomics platforms. This figure compares the precision and accuracy of feature extraction across platforms, categorized into four levels of variance: excellent, good, moderate, and poor. RD, relative difference.

Figure 4 illustrates the RD^6^ values for all evaluated features, categorized into four levels of variance. This diagram aids in the direct comparison of the precision and accuracy of feature extraction across different platforms, shedding light on the effectiveness of each platform's computational algorithms. In the analysis of the three feature categories, results for the morphology category showed that LIFEx and PyRadiomics achieved an excellent rating (RD ≤ 1%) for 100% of features. In contrast, CERR demonstrated 23% excellence (3 out of 13 features) and 30% good. In the statistics/histogram category, a higher RD value was observed, with 100% of the features rated excellent for LIFEx, 82% (23 out of 28 features) for PyRadiomics, and 87% (34 out of 39 features) for CERR. The texture feature category revealed that LIFEx maintained 100% excellence, whereas PyRadiomics and CERR exhibited 69% (56 out of 81 features) and 98% (117 out of 120 features) excellence, respectively.

Table 3 presents a comprehensive correlation analysis between the platforms, focusing on 64 common features. It lists the CCC and PCC values, indicating a high level of consistency in feature extraction with values > 0.999.

**TABLE 3:** CCC and PCC analyses for common features across radiomics software platforms.

Inter‐platforms test	LIFEx	PyRadiomics	CERR	LIFEx	PyRadiomics	CERR
LIFEx	1	0.999	0.999	1	0.999	0.999
PyRadiomics	0.999	1	0.999	0.999	1	0.999
CERR	0.999	0.999	1	0.999	0.999	1

Abbreviations: CCC, concordance correlation coefficient; CERR, computational environment for radiological research; PCC, Pearson correlation coefficient.

* *p*‐values of all coefficients are < 0.05.

## 4 DISCUSSION

This study comprehensively examined the reproducibility of radiomics feature extraction across three IBSI‐compliant platforms, namely, LIFEx, CERR, and PyRadiomics, using a digital phantom as the benchmark. Our study aimed to investigate the consistency of radiomics feature extraction, underscoring the critical need for standardized computational algorithms and mathematical definitions to improve the reliability and clinical utility of radiomics. By analyzing 215 IBSI‐standardized features, categorized into morphology, statistics/histogram, and texture, our study used a rigorous statistical framework to evaluate reproducibility across these platforms. As highlighted in Tables S1–S3, the inclusion of IBSI reference values alongside the extracted feature data establishes a transparent framework for evaluating reproducibility across platforms. The detailed comparison of feature values demonstrates both the strengths and limitations of current IBSI‐compliant methods, emphasizing the necessity for ongoing standardization efforts to enhance cross‐platform consistency in radiomics research.

The analysis of the morphology category (Table 2, Figure 3) identified that only five features—Volume, Surface Area, Surface‐to‐Volume Ratio, Maximum 3D Diameter, and Sphericity—were consistently extracted across all platforms. These features are critical for understanding the geometric and structural properties of ROIs in radiomics studies. Their consistent reproducibility highlights their reliability and importance in both clinical and research applications, as their values are predominantly unaffected by platform‐specific computational variations. Volume and Surface Area quantify the size and extent of a structure, while the Surface‐to‐Volume Ratio provides insights into shape complexity and compactness, potentially reflecting pathological irregularities or biological processes.^8^, ^24^ Maximum 3D Diameter is a robust measure for cancer staging and assessing tumor spread,^24^ while Sphericity quantifies how closely a structure approximates a perfect sphere, aiding in the differentiation of benign and malignant lesions. Malignant tumors often exhibit irregular and invasive growth patterns, making Sphericity a valuable metric in clinical evaluations.^25^ The reproducibility of these morphological features across multiple software platforms underscores their potential as reliable quantitative imaging biomarkers. Their inclusion in radiomics signatures enhances the performance of diagnostic, prognostic, and predictive models in various clinical settings.^26^, ^27^

Among the 13 morphological features evaluated for CERR, three were rated excellent, four good, four moderate, and two poor. These variations stem from CERR's unique 3D calculation algorithm, which differs from the voxel‐based approaches used by LIFEx and PyRadiomics.^15^, ^23^ While CERR's 3D algorithm increases the granularity of morphological assessments, it also introduces sensitivity to segmentation boundary definitions, voxel discretization, and interpolation settings.^28^ Additionally, the algorithm's reliance on approximations and discretization increases the likelihood of errors that affect the accuracy of morphological feature calculations.^15^, ^29^ Harmonized computational algorithms and comprehensive documentation of methodological details are essential for consistent feature extraction and improved inter‐platform agreement.

The selection of feature extraction parameters is essential for ensuring reliability and reproducibility in radiomics. Standardizing these parameters minimizes variability and enhances analytical robustness across platforms and institutions. For example, the bin size directly influences image intensity quantization; a bin size of 1 provides high‐resolution insights but increases sensitivity to noise. Similarly, the interpolation method affects sampling artifacts and distortions, with B‐spline interpolation offering smoother resampling compared to nearest‐neighbor or linear methods. The GLCM distance parameter, typically set to 1, is critical for texture analysis as it captures local heterogeneity among neighbor pixels. Additionally, resampling pixel spacing often applied to standardize image dimensions, can alter feature values by modifying resolution. Preserving the original resolution avoids changes to morphological and texture integrity. Adhering to IBSI‐recommended parameter settings strengthens reproducibility, enabling more reliable and clinically meaningful outcomes across platforms.

In the analysis of the statistics/histogram category, 27 of 50 features were identified as common features across platforms. Notably, LIFEx demonstrated 100% of its features within the excellent group, according to the RD^6^ analysis, which also showed the highest number of extracted features. Among these common features, a particular distinction was observed in kurtosis, with only PyRadiomics exhibiting a significant deviation. This deviation is attributed to differences in the mathematical calculations used by the platforms. IBSI, CERR, and LIFEx adhere to Equation 1 for the kurtosis calculation:

(1) $$\mathrm{Kurtosis}=\frac{\mu^{4}}{\sigma^{4}}-3$$

where *μ* represents the central moment and *σ* the standard deviation. However, PyRadiomics uses Equation 2:

(2) $$\mathrm{Kurtosis}=\frac{\mu^{4}}{\sigma^{4}}$$

This variation in mathematical formulations highlights subtle but meaningful differences in how features are calculated across platforms.^17^

In the Texture category, only 32 out of 136 features were common across all platforms despite the extensive number of features analyzed. This limited overlap stems from differences in calculation methods employed by the platforms. For example, the GLCM feature group, comprising 50 features, was calculated using two distinct methods: averaged and merged. Specifically, LIFEx utilizes a “merged” method for GLCM calculation, summing directional matrices into one, whereas PyRadiomics employs an “averaged” approach, computing matrices for each direction and averaging them. While both adhere to the core GLCM concept, these aggregation strategies inevitably yield variations in feature values, underscoring the necessity for standardized GLCM calculation guidelines. Similarly, in the GLRLM group, consisting of 32 features, LIFEx supports only the averaged method despite benchmarks existing for both calculation methods. Additionally, certain feature groups were exclusively calculated by specific platforms, further contributing to the limited number of common features. For instance, the GLSZM features, totaling 16, were calculated solely by PyRadiomics, while the NGLDM features, totaling 17, were computed only by CERR. In contrast, all five features analyzed from the NGTDM were common across platforms, each exhibiting a RD within 1.0%, demonstrating high accuracy and consistency.

The observed discrepancies in the total number of extracted features (e.g., 41, 39, and 28 in the Statistics/Histogram category; 55, 120, and 81 in the Texture category) primarily reflect each platform's distinct methods of defining, grouping, or naming features rather than outright incompatibility. As radiomics software continues to evolve and as the IBSI refines its guidelines, the set of shared or “common” features across platforms will likely expand, enhancing cross‐platform reproducibility. This highlights the critical role of community‐driven standardization efforts and diligent software updates, which, over time, should bridge existing gaps in feature definitions and advance the clinical utility of radiomics.

The CCC and PCC for common radiomics features extracted from LIFEx, PyRadiomics, and CERR demonstrated very strong correlations (coefficients > 0.999, *p* < 0.05), indicating consistent results for common features despite variations in individual radiomics feature values. The observed RD values, categorized as excellent (RD ≤ 1%), good (1% < RD ≤ 5%), moderate (5% < RD ≤ 10%), and poor (RD > 10%), can estimate a feature's reliability in tasks such as tumor characterization or treatment response prediction. Features classified as “excellent” or “good” are more likely to exhibit consistent performance across medical centers, while those rated “moderate” or “poor” require further evaluation before clinical implementation. These findings underscore the necessity of improving the precision of radiomics software and adhering to standardized guidelines, such as the IBSI, to ensure the reliability and interpretability of results. Such efforts are essential for facilitating the integration of radiomics into personalized medicine.

Compared with the studies by Lei et al., Fornacon‐Wood et al., and Paquier et al., our findings align with and diverge from previous observations on feature reproducibility and the role of IBSI compliance in ensuring consistent feature extraction.^18^, ^19^, ^22^ Lei et al. used IBSI benchmarks across various platforms and reported generally satisfactory agreement for most features but mentioned significant variations in morphological features, highlighting the challenges of feature standardization.^18^ Fornacon‐Wood et al. pointed out that radiomics feature reliability significantly increased within IBSI‐compliant platforms, although discrepancies owing to unharmonized calculation settings could compromise reliability.^22^ The exploration of feature value concordance across IBSI‐compliant software by Paquier et al. revealed that compliance alone did not eliminate discrepancies in feature values, potentially affecting radiomics model performance and generalizability.^19^ Lei et al. and Paquier et al. considered this necessity, highlighting the ongoing challenges despite IBSI compliance and calling for continued quality assurance and standardization efforts.^18^, ^19^

Our findings, together with those of these studies, emphasize the critical role that compliance with the IBSI guidelines plays in making the extraction of features more reliable. However, we still face significant challenges, particularly regarding the shape features and differences arising from various calculation methods or software versions. This insight emphasizes the ongoing need for efforts toward greater standardization, including the alignment of feature definitions and calculation methods and clear documentation of software versions and settings to guarantee the reliability and reproducibility of radiomics research. In addition, the integration of these findings into clinical workflows has the potential to move radiomics beyond theoretical innovation and deliver practical benefits to patients. This integration could help bridge the gap between novel algorithmic developments and their application in routine clinical practice, facilitating advancements in personalized medicine and enhancing diagnostic and predictive models. Our study focused on harmonizing feature calculation methods and definitions across platforms to enhance the reliability and clinical applicability of radiomics based on a detailed analysis using a digital phantom.

## 5 CONCLUSION

This study comprehensively evaluated radiomics feature reproducibility across three IBSI‐compliant platforms, demonstrating high consistency among common features while identifying unique platform‐specific features. These findings highlight the urgent need for standardized computational algorithms and mathematical definitions to ensure the reliability and clinical applicability of radiomics. By thoroughly analyzing feature categories such as morphology, statistics/histogram, and texture, this study underscores the critical importance of harmonizing calculation methods and definitions. Achieving such standardization is essential for advancing radiomics as a robust and reliable tool in clinical decision‐making and personalized medicine.

## AUTHOR CONTRIBUTIONS

*Concept and design*: Heesoon Sheen: *Acquisition, analysis, or interpretation of data*: All authors. *Drafting of the manuscript*: Han‐Back Shin, Heesoon Sheen. *Critical review of the manuscript for important intellectual content*: Hyun Ju Kim, Jang‐Hoon Oh. *Statistical analysis*: Han‐Back Shin, Heesoon Sheen, Jang‐Hoon Oh. *Administrative, technical, or material support*: Han‐Back Shin, Hyun Ju Kim, Jang‐Hoon Oh, Young Eun Choi, Kihoon Sung. *Supervision*: Hyun Ju Kim.

## CONFLICT OF INTEREST STATEMENT

The authors declare no conflicts of interest.

## Supporting information



Supporting information
