# Supplementary material for: Evaluating feature extraction reproducibility across image biomarker standardization initiative‐compliant radiomics platforms using a digital phantom
Source: J Appl Clin Med Phys. 2025 May 12;26(7):e70110. doi: 10.1002/acm2.70110 (PMC12256664; doi:10.1002/acm2.70110)
Supplement: Supplementary file 1 — Supporting information [file ACM2-26-e70110-s001.docx]

This supplementary table provides a detailed comparison of radiomics features analyzed across three IBSI-compliant platforms (LIFEx, PyRadiomics, and CERR). Features highlighted in yellow represent common features that were consistently extracted across all platforms, demonstrating their reliability and reproducibility under IBSI standards.

Supplementary Table 1. Detailed list of Morphology category features

| Feature Name (n=29) | IBSI reference value | LIFEx | PyRadiomics | CERR |
| --- | --- | --- | --- | --- |
| Volume (mesh) | 566 | 566.3 | 566.3 | - |
| Volume (voxel counting)***** | 592 | 592.0 | 592.0 | 592 |
| Surface area (mesh)* | 388 | 388.1 | 388.1 | 381.1 |
| Surface to volume ratio***** | 0.698 | 0.698 | 0.698 | 0.644 |
| Compactness 1 | 0.0411 | 0.041 | - | 0.045 |
| Compactness 2 | 0.599 | 0.599 | - | 0.716 |
| Spherical disproportion | 1.19 | 1.186 | - | 1.12 |
| Sphericity***** | 0.843 | 0.843 | 0.843 | 0.895 |
| Asphericity | 0.186 | 0.186 | - | - |
| Centre of mass shift | 0.672 | 0.672 | - | - |
| Maximum 3D diameter***** | 13.1 | 13.12 | 13.11 | 11.66 |
| Major axis length | 11.4 | - | 11.3 | 11.3 |
| Minor axis length | 9.31 | - | 9.25 | 9.21 |
| Least axis length | 8.54 | - | 8.48 | 8.42 |
| Elongation | 0.816 | - | 0.816 | 0.818 |
| Flatness | 0.749 | - | 0.749 | 0.749 |
| Volume density (AABB) | 0.869 | - | - | - |
| Area density (AABB) | 0.866 | - | - | - |
| Volume density (OMBB) | ** | - | - | - |
| Area density (OMBB) | ** | - | - | - |
| Volume density (AEE) | 1.17 | - | - | - |
| Area density (AEE) | 1.36 | - | - | - |
| Volume density (MVEE) | ** | - | - | - |
| Area density (MVEE) | ** | - | - | - |
| Volume density (convex hull) | 0.961 | - | - | - |
| Area density (convex hull) | 1.03 | - | - | - |
| Integrated intensity | 1200 | 1195 | - | - |
| Moran's I index | 0.0397 | - | - | - |
| Geary's C measure | 0.974 | - | - | - |

* Common features extracted across the three radiomics platforms

** Features not yet standardized by IBSI

Supplementary Table 2. Detailed list of Stastic and Histogram category features

| Feature Name (n=50) | IBSI reference value | LIFEx | PyRadiomics | CERR |
| --- | --- | --- | --- | --- |
| Local intensity peak+ | 2.6 | - | - | - |
| Global intensity peak+ | 3.1 | - | - | - |
| Mean*+ | 2.15 | 2.15 | 2.15 | 2.15 |
| Variance*+ | 3.05 | 3.05 | 3.05 | 3.05 |
| Skewness*+ | 1.08 | 1.08 | 1.08 | 1.08 |
| (Excess) kurtosis*+ | -0.355 | -0.355 | 2.65 | -0.355 |
| Median*+ | 1 | 1 | 1 | 1 |
| Minimum*+ | 1 | 1 | 1 | 1 |
| 10th percentile*+ | 1 | 1 | 1 | 1 |
| 90th percentile*+ | 4 | 4 | 4 | 4 |
| Maximum*+ | 6 | 6 | 6 | 6 |
| Interquartile range*+ | 3 | 3 | 3 | 3 |
| Range*+ | 5 | 5 | 5 | 5 |
| Mean absolute deviation*+ | 1.55 | 1.55 | 1.55 | 1.55 |
| Robust mean absolute deviation*+ | 1.11 | 1.11 | 1.11 | 1.11 |
| Median absolute deviation+ | 1.15 | 1.15 | - | 1.15 |
| Coefficient of variation+ | 0.812 | 0.812 | - | 0.81 |
| Quartile coefficient of dispersion+ | 0.6 | 0.6 | - | 0.6 |
| Energy*+ | 567 | 567 | 567 | 567 |
| Root mean square*+ | 2.77 | 2.77 | 2.77 | 2.77 |
| Mean*++ | 2.15 | 2.15 | 2.15 | 2.15 |
| Variance*++ | 3.05 | 3.05 | 3.05 | 3.05 |
| Skewness++ | 1.08 | 1.08 | - | 1.08 |
| (Excess) kurtosis*++ | -0.355 | -0.355 | 2.65 | -0.355 |
| Median*++ | 1 | 1 | 1 | 1 |
| Minimum*++ | 1 | 1 | 1 | 1 |
| 10th percentile*++ | 1 | 1 | 1 | 1 |
| 90th percentile*++ | 4 | 4 | 4 | 4 |
| Maximum*++ | 6 | 6 | 6 | 6 |
| Mode++ | 1 | 1 | - | - |
| Interquartile range*++ | 3 | 3 | 3 | 3 |
| Range++ | 5 | 5 | - | 5 |
| Mean absolute deviation*++ | 1.55 | 1.55 | 1.55 | 1.55 |
| Robust mean absolute deviation*++ | 1.11 | 1.11 | 1.11 | 1.11 |
| Median absolute deviation++ | 1.15 | 1.15 | - | 1.15 |
| Coefficient of variation++ | 0.812 | 0.812 | - | 0.812 |
| Quartile coefficient of dispersion++ | 0.6 | 0.6 | - | 0.6 |
| Entropy*++ | 1.27 | 1.27 | -3.20E-16 | -1.64E-16 |
| Uniformity++ | 0.512 | 0.512 | 1 | - |
| Maximum histogram gradien++ | 8 | 8 | - | - |
| Maximum histogram gradient intensity++ | 3 | 3 | - | - |
| Minimum histogram gradient++ | -50 | -50 | - | - |
| Minimum histogram gradient intensity | 1 | 1 | - | - |
| Volume fraction at 10% intensity++ | 0.324 | - | - | 0.324 |
| Volume fraction at 90% intensity++ | 0.0946 | - | - | 0.0946 |
| Intensity at 10% volume++ | 5 | - | - | 5.11 |
| Intensity at 90% volume++ | 2 | - | - | 2.65 |
| Volume fraction difference between 10% and 90% intensity++ | 0.23 | - | - | - |
| Intensity difference between  10% and 90% volume++ | 3 | - | - | - |
| Area under the IVH curve++ | ** | - | - | - |

* Common features extracted across the three radiomics platforms

** Features not yet standardized by IBSI

+ Stastic features, ++ Histogram features

Supplementary Table 3. Full list of Texture features used in this study.

| Feature Name | IBSI reference value | LIFEx | PyRadiomics | CERR |
| --- | --- | --- | --- | --- |
| GLCM (3D averaged algorithm, n=25) | | | | |
| Joint maximum | 0.50 | - | - | 0.50 |
| Joint average | 2.14 | - | 2.14 | 2.14 |
| Joint variance | 3.10 | - | - | 3.10 |
| Joint entropy | 2.40 | - | 2.40 | 2.40 |
| Difference average | 1.43 | - | 1.43 | 0.00 |
| Difference variance | 3.06 | - | 3.06 | 3.06 |
| Difference entropy | 1.56 | - | 1.56 | 1.56 |
| Sum average | 4.29 | - | 4.29 | 4.29 |
| Sum variance | 7.07 | - | - | 7.07 |
| Sum entropy | 1.92 | - | 1.92 | 1.92 |
| Angular second moment | 0.30 | - | - | 0.30 |
| Contrast | 5.32 | - | 5.32 | 5.32 |
| Dissimilarity | 1.43 | - | - | 1.43 |
| Inverse difference | 0.68 | - | - | 0.68 |
| Normalised inverse difference | 0.85 | - | - | 0.85 |
| Inverse difference moment | 0.62 | - | - | 0.62 |
| Normalised inverse difference moment | 0.90 | - | - | 0.90 |
| Inverse variance | 0.06 | - | 0.06 | 0.06 |
| Correlation | 0.16 | - | 0.16 | 0.16 |
| Autocorrelation | 5.06 | - | 5.06 | 5.06 |
| Cluster tendency | 7.07 | - | 7.07 | 7.07 |
| Cluster shade | 16.60 | - | 16.60 | 16.60 |
| Cluster prominence | 145.00 | - | 145.00 | 145.00 |
| Information correlation 1 | -0.16 | - | - | -0.16 |
| Information correlation 2 | 0.52 | - | - | 0.52 |
| GLCM (3D merged algorithm, n=25) | | | | |
| Joint maximum | 0.51 | 0.51 | - | 0.51 |
| Joint average | 2.15 | 2.15 | - | 2.15 |
| Joint variance | 3.13 | 3.13 | - | 3.13 |
| Joint entropy | 2.57 | 2.57 | - | 2.57 |
| Difference average | 1.38 | 1.38 | - | 0.00 |
| Difference variance | 3.21 | 3.22 | - | 3.21 |
| Difference entropy | 1.64 | 1.64 | - | 1.64 |
| Sum average | 4.30 | 4.30 | - | 4.30 |
| Sum variance | 7.41 | 7.41 | - | 7.41 |
| Sum entropy | 2.11 | 2.12 | - | 2.11 |
| Angular second moment | 0.29 | 0.29 | - | 0.29 |
| Contrast | 5.12 | 5.12 | - | 5.12 |
| Dissimilarity | 1.38 | 1.38 | - | 1.38 |
| Inverse difference | 0.69 | 0.69 | - | 0.69 |
| Normalised inverse difference | 0.86 | 0.86 | - | 0.86 |
| Inverse difference moment | 0.63 | 0.63 | - | 0.63 |
| Normalised inverse difference moment | 0.90 | 0.90 | - | 0.90 |
| Inverse variance | 0.06 | 0.06 | - | 0.06 |
| Correlation | 0.18 | 0.18 | - | 0.18 |
| Autocorrelation | 5.19 | 5.19 | - | 5.19 |
| Cluster tendency | 7.41 | 7.41 | - | 7.41 |
| Cluster shade | 17.40 | 17.40 | - | 17.40 |
| Cluster prominence | 147.00 | 147.00 | - | 147.50 |
| Information correlation 1 | -0.03 | - | - | -0.03 |
| Information correlation 2 | 0.27 | - | - | 0.27 |
| GLRLM (3D averaged algorithm, n=16) | | | | |
| Short runs emphasis* | 0.705 | 0.705 | 0.705 | 0.705 |
| Long runs emphasis* | 3.060 | 3.061 | 3.061 | 3.061 |
| Low grey level run emphasis* | 0.603 | 0.603 | 0.603 | 0.603 |
| High grey level run emphasis* | 9.700 | 9.698 | 9.698 | 9.698 |
| Short run low grey level emphasis* | 0.352 | 0.352 | 0.352 | 0.352 |
| Short run high grey level emphasis* | 8.540 | 8.540 | 8.540 | 8.540 |
| Long run low grey level emphasis* | 2.390 | 2.391 | 2.391 | 2.391 |
| Long run high grey level emphasis* | 17.600 | 17.566 | 17.566 | 17.566 |
| Grey level non-uniformity* | 21.800 | 21.776 | 21.776 | 21.776 |
| Normalised grey level non-uniformity | 0.430 |  | 0.430 | 0.430 |
| Run length non-uniformity* | 26.900 | 26.853 | 26.853 | 26.853 |
| Normalised run length non-uniformity | 0.513 |  | 0.513 | 0.513 |
| Run percentage* | 0.680 | 0.680 | 0.680 | 0.680 |
| Grey level variance | 3.460 |  | 3.465 | 3.465 |
| Run length variance | 0.574 |  | 0.574 | 0.574 |
| Run entropy | 2.430 |  | 2.432 | 2.432 |
| GLRLM (3D merged algorithm, n=16) | | | | |
| Short runs emphasis | 0.73 | - | 0.71 | 0.73 |
| Long runs emphasis | 2.76 | - | 3.06 | 2.76 |
| Low grey level run emphasis | 0.61 | - | 0.60 | 0.61 |
| High grey level run emphasis | 9.64 | - | 9.70 | 9.64 |
| Short run low grey level emphasis | 0.37 | - | 0.35 | 0.37 |
| Short run high grey level emphasis | 8.67 | - | 8.54 | 8.67 |
| Long run low grey level emphasis | 2.16 | - | 2.39 | 2.16 |
| Long run high grey level emphasis | 15.60 | - | 17.57 | 15.63 |
| Grey level non-uniformity | 281.00 | - | 21.78 | 281.28 |
| Normalised grey level non-uniformity | 0.43 | - | 0.43 | 0.43 |
| Run length non-uniformity | 328.00 | - | 26.85 | 327.72 |
| Normalised run length non-uniformity | 0.50 | - | 0.51 | 0.50 |
| Run percentage | 0.68 | - | 0.68 | 8.84 |
| Grey level variance | 3.48 | - | 3.46 | 3.48 |
| Run length variance | 0.60 | - | 0.57 | 0.60 |
| Run entropy | 2.62 | - | 2.43 | 2.62 |
| GLSZM (n=16) | | | | |
| Small distance emphasis* | 0.26 | 0.26 | 0.26 | 0.26 |
| Large distance emphasis* | 550.00 | 550.00 | 550.00 | 550.00 |
| Low grey level emphasis* | 0.25 | 0.25 | 0.25 | 0.25 |
| High grey level emphasis* | 15.60 | 15.60 | 15.60 | 15.60 |
| Small distance low grey level emphasis* | 0.03 | 0.03 | 0.03 | 0.03 |
| Small distance high grey level emphasis* | 2.76 | 2.76 | 2.76 | 2.76 |
| Large distance low grey level emphasis* | 503.00 | 502.79 | 502.79 | 502.79 |
| Large distance high grey level emphasis* | 1490.00 | 1494.60 | 1494.60 | 1494.60 |
| Grey level non-uniformity* | 1.40 | 1.40 | 1.40 | 1.40 |
| Normalised grey level non-uniformity* | 0.28 | 0.28 | 0.28 | 0.28 |
| Zone distance non-uniformity* | 1.00 | 1.00 | 1.00 | 1.00 |
| Normalised zone distance non-uniformity* | 0.20 | 0.20 | 0.20 | 0.20 |
| Zone percentage* | 0.07 | 0.07 | 0.07 | 0.07 |
| Grey level variance* | 2.64 | 2.64 | 2.64 | 2.64 |
| Zone distance variance* | 331.00 | 330.96 | 330.96 | 330.96 |
| Zone distance entropy* | 2.32 | 2.32 | 2.32 | 2.32 |
| Distance Zone Matrix (n=16) | | | | |
| Small distance emphasis | 1.00 | - | 0.04 | - |
| Large distance emphasis | 1.00 | - | 109.00 | - |
| Low grey level emphasis | 0.25 | - | 0.69 | - |
| High grey level emphasis | 15.60 | - | 7.66 | - |
| Small distance low grey level emphasis | 0.25 | - | 0.01 | - |
| Small distance high grey level emphasis | 15.60 | - | 0.74 | - |
| Large distance low grey level emphasis | 0.25 | - | 102.45 | - |
| Large distance high grey level emphasis | 15.60 | - | 234.99 | - |
| Grey level non-uniformity | 1.40 | - | 37.92 | - |
| Normalised grey level non-uniformity | 0.28 | - | - | - |
| Zone distance non-uniformity | 5.00 | - | 4.86 | - |
| Normalised zone distance non-uniformity | 1.00 | - | 0.07 | - |
| Zone percentage | 0.07 | - | - | - |
| Grey level variance | 2.64 | - | 3.05 | - |
| Zone distance variance | 0.00 | - | 22.06 | - |
| Zone distance entropy | 1.92 | - | 4.40 | - |
| NGTDM (5) | | | | |
| Coarseness* | 0.03 | 0.03 | 0.03 | 0.03 |
| Contrast* | 0.58 | 0.58 | 0.58 | 0.58 |
| Busyness* | 6.54 | 6.54 | 6.54 | 6.54 |
| Complexity* | 13.50 | 13.54 | 13.54 | 13.54 |
| Strength* | 0.76 | 0.76 | 0.76 | 0.76 |
| NGLDM (17) | | | | |
| Low dependence emphasis | 0.05 | - | - | 0.05 |
| High dependence emphasis | 109.00 | - | - | 109.00 |
| Low grey level count emphasis | 0.69 | - | - | 0.69 |
| High grey level count emphasis | 7.66 | - | - | 7.66 |
| Low dependence low grey level emphasis | 0.01 | - | - | 0.01 |
| Low dependence high grey level emphasis | 0.74 | - | - | 0.74 |
| High dependence low grey level emphasis | 102.00 | - | - | 102.45 |
| High dependence high grey level emphasis | 235.00 | - | - | 234.99 |
| Grey level non-uniformity | 37.90 | - | - | 37.92 |
| Normalised grey level non-uniformity | 0.51 | - | - | 0.51 |
| Dependence count non-uniformity | 4.86 | - | - | 4.86 |
| Normalised dependence count non-uniformity | 0.07 | - | - | 0.07 |
| Dependence count percentage | 1.00 | - | - | 1.00 |
| Grey level variance | 3.05 | - | - | 3.05 |
| Dependence count variance | 22.10 | - | - | 22.06 |
| Dependence count entropy | 4.40 | - | - | 4.40 |
| Dependence count energy | 0.05 | - | - | 0.05 |

GLCM: Gray Level Co-occurrence Matrix

GLRLM: Gray Level Run Length Matrix

GLSZM: Gray Level Size Zone Matrix

NGTDM: Neighboring Gray Tone Difference Matrix

GLCM, GLRLM values are calculated using two methods: averaged and merged calculation methods.

* Common features extracted across the three radiomics platforms
